# Prevalence and Characteristics of Medical Student Mistreatment in Lebanon

**DOI:** 10.3389/ijph.2024.1606710

**Published:** 2024-07-04

**Authors:** Ahmad El Nouiri, Sarah El Kassem, Zeina Al Maaz, Yasser Alhajj, Alhareth Al Moussawi, Ahmad El Yaman, Hussein El Hajjar, Mohannad Abdallah, Gaith Assi, Mohamad Houri, Bilal Azakir

**Affiliations:** ^1^ Faculty of Medicine, Beirut Arab University, Beirut, Lebanon; ^2^ Lebanese American University, Beirut, Lebanon

**Keywords:** student mistreatment, prevalence, Lebanon, mistreatment characteristics, patients

## Abstract

**Objective:**

This study aimed to determine the prevalence of medical student mistreatment in Lebanon, the framework of the incidents, and the extent of students’ knowledge on mistreatment characteristics.

**Methods:**

This is a cross-sectional study conducted using an online-based survey among medical students who have performed clinical rotations in Lebanon.

**Results:**

Out of 300 respondents, 48.7% reported being subjected to mistreatment during clinical practice, which was significantly associated with gender, type of university, and family income. The two most common sources of mistreatment were patients and their families/friends (77.4%), and attending physicians (52.7%), followed by residents (49.3%). Students mostly chose to be passive and pacifying. Additionally, 64.7% of students stated they were not trained about the ideal way to handle these incidents.

**Conclusion:**

This study showed that medical student mistreatment is highly prevalent in Lebanon. It also highlighted the lack of proper education on mistreatment characteristics and the necessity for investigating its effects.

## Introduction

As a major part of a physician’s education, the World Federation of Medical Education (WFME) considers Clinical skills acquired from clinical facilities one of the seven criteria for establishing a high-quality medical school [1]. The interaction between patient and student-mentor relationship helps medical students in develop their essential skills of diagnosis, communication, and professionalism.

Unfortunately, medical students are subject to mistreatment during clinical rotations from different sources. The Association of American Medical Colleges defines mistreatment, as a behavior that shows disrespect for the dignity of others and unreasonably interferes with the learning process. It includes many domains such as physical mistreatment, abusive language, sexual harassment, discrimination, and others [2–6].

One source of mistreatment is the medical educators who heavily rely on asking students provocative questions, hoping that this will the concepts in their minds [7]. Many educators, however, take this method to the extreme, resulting in belittlement and mistreatment incidents. Additionally, patients and their families are another source of mistreatment. Patients may feel uncomfortable with training students getting in contact with them or getting involved in their care. This feeling may be due to underestimation of the role of clinical exposure in developing students’ medical competencies and a lack of trust in them.

Students’ mistreatment can be affected by many factors, such as educational background, culture, and socioeconomic status. Mistreatment has a multitude of negative effects on students’ psychology and personalities, since multiple studies have established an association with depression, desire to drop out of school, low career satisfaction, decreased confidence in clinical abilities, symptoms of posttraumatic stress disorder, increased burnout, alcohol abuse, and suicidality [8–14]. Moreover, medical student mistreatment can negatively impact the patient outcomes [15].

Several studies aimed to determine the prevalence of medical students' mistreatment worldwide. A meta-analysis was conducted on 51 studies addressing harassment and discrimination in medical training and revealed that 59.4% of medical residents reported experiencing at least one form of harassment or discrimination during their training, with attending physicians being the most commonly cited source (34.4%) and patients or their families, the second most common (21.9%) [16]. Another study conducted in the United States reported that 15% of mistreatment occurred among pediatric residents, 67% of which originated from patients’ families [17]. Regrettably, 50% of the mistreated residents did not know how to respond to the situation, and 25% of them did not believe any action would be taken by the hospital leadership if they had reported the incident [17].

Little data is present about the prevalence of mistreatment of medical students in the Mediterranean region, with a complete lack of data in Lebanon. Thus, a study about the prevalence of medical students' mistreatment and the extent of students’ knowledge on the proper way to handle it is essential to efficiently manage this issue.

## Methods

### Study Design and Participants

This is a descriptive, survey-based, cross-sectional study conducted across medical schools in Lebanon. The population included medical students in Lebanon who had been exposed to clinical rotations. There were no exclusion criteria. The survey was distributed online using a snowball sampling method among all medical students in Lebanon. The calculated sample size was determined to be a minimum of 292 for a 95% confidence interval with a 5% margin of error. The sample size was calculated based on an estimation of 1,212 clerkship students enrolled at the eight Faculties of Medicine in Lebanon [18].

### Settings

A self-administered survey was adopted from previous studies and adjusted to meet the study objectives [19]. The survey was piloted on 20 students to assess questions comprehension and integrity of the questions. The survey was designed using Google Forms and distributed using snowball technique to medical students. It was divided into four parts. The first part was about sociodemographic information of the students. The second part was about the prevalence of mistreatment among students. The third part was about the characteristics of mistreatment episode by patients or their families/friends. The fourth part was about students’ knowledge of mistreatment characteristics. The survey contained 35 questions and took about 10 min to complete.

### Ethical Aspects

This study was approved by the Institutional Review Board of Beirut Arab University under the number 0113-M-R-0495. The study followed the guidelines of the Declaration of Helsinki on the conduct of human research. All data collected were de-identified, gathered, and checked by the principal investigator. The adopted consent form follows the university IRB requirements. This research study is considered to be of minimal risk; the risks associated with this study are the same as those participants face every day.

### Patient and Public Involvement Statement

A pilot study was conducted to identify medical students' exposure to mistreatment and the survey questions’ validity and comprehensibility. Otherwise, participants were not involved in the study’s conceptualization.

### Statistical Analysis

Statistical Package for the Social Sciences (IBM SPSS, version 25) was used for data entry, cleaning, management, and analyses. Descriptive analyses were carried out by calculating the mean and standard deviation for continuous variables, and numbers and percentages for categorical ones. Inferential statistics, mainly bivariate analyses, were carried out by using the Chi-square test for categorical variables, and associations were measured using Crammer’s V test. A Crammer’s V coefficient: between 0 and 0.1 was considered negligible, between 0.1 and 0.2 weak, between 0.2 and 0.4 moderate, between 0.4 and 0.6 relatively strong, between 0.6 and 0.8 strong, and between 0.8 and 1 very strong. When applicable, Fisher’s exact test was used. A *p*-value < 0.05 was considered significant.

## Results

### Participants’ Characteristics

A total of 300 participants responded to the survey, with a mean age of 23.51 ± 1.54. The majority were Lebanese, single, and had no children, and 51.3% were females. Participants were mainly living in Beirut (42.7%) and were enrolled in private universities (80.7%), with half of them reporting an insufficient family income (Table 1).

**TABLE 1:** Socio-demographic information of participants S.D: Standard Deviation (Beirut, Lebanon. 2024).

	Mean ± S.D
Age	23.51 ± 1.54	
	Number	Percentage
Gender (300)		
Male	146	48.7
Female	154	51.3
Nationality (300)		
Lebanese	294	98
Non-Lebanese	6	2
Place of Residence (300)		
North-Lebanon	31	10.3
Beirut	128	42.7
Bekaa-Baalbeck	21	7
Mount-Lebanon	78	26
South-Lebanon	42	14
Marital Status (300)		
Single	284	94.7
Married	16	5.3
Children (300)		
Yes	6	2
No	294	98
Family Income (187)		
Insufficient	95	50.8
Sufficient	92	49.20
Type of University (300)		
Public	58	19.3
Private	242	80.7

### Mistreatment Prevalence and Sources

The prevalence of students’ mistreatment during their clinical practice was 48.7%, with 51.4% of participants stating that they faced a form of mistreatment at least 2–5 times and 6.8% at least 5–10 times. The most common sources of mistreatment reported were patients and their families/friends (77.4%) followed by attending physicians (52.7%) and residents (49.3%) (Table 2).

**TABLE 2:** Sources and frequency of mistreatment (Beirut, Lebanon. 2024).

	Number	Percentage
Have you been mistreated during clinical practice? (300)		
Yes	146	48.7
No	154	51.3
Frequency of Mistreatment (146)		
Once	46	31.5
2–5 times	75	51.4
5–10 times	10	6.8
More than 10 times	15	10.3
Mistreatment from other sources (146)^a^		
Attending Physicians	77	52.7
Residents	72	49.3
Patients and their families/friends	113	77.4
Nurses	65	44.5
Other Medical Students	28	19.2
Ancillary Staff (Cooks, Cleaners)	13	8.9
Frequency of Mistreatment by Patients, Family/Friends (113)		
Low	81	71.7
Moderate	19	16.8
High	13	11.5

^a^ Multiple response questions.

### Mistreatment Incident Characteristics

The majority of instances occurred in private hospitals/healthcare centers (66.4%), with almost half of them taking place in wards (48.7%). Last year’s medical students were more exposed to mistreatment (64.6%), and around three-quarters of occurrences were during internal medicine rotations. The incidents happened mostly during history taking (52.2%) and mainly were classified as emotional mistreatment (55.8%). The incident happened mainly when students were alone (40.7%). When asking participants about how they dealt with the incidents, 39.8% of the mistreated students chose to pacify themselves, and 77.9% reported feeling angry or sad. Regarding the way the situations were addressed, a superior intervened in 43.4% of the instances. Most of the cases were resolved (85.8%), and students were mostly satisfied with the resolutions (65.5%). While the majority of students claimed the incidents did not affect their academic performance (61.1%), 35.4% said the incident had a negative impact on their academic performance (Table 3).

**TABLE 3:** Information about mistreatment incident from patients, families/friends (Beirut, Lebanon. 2024).

	Number	Percentage
Type of Institution where the incident happened (113)		
Public Hospital/Healthcare Center	38	33.6
Private Hospital/Healthcare Center	75	66.4
Type of Mistreatment (113)		
Emotional	63	55.8
Mental-Psychology	23	20.4
Physical	27	23.9
State of the Student (113)		
History Taking	59	52.2
Physical Examination	40	35.4
Intervention	14	12.4
Place of Incident (113)		
Floor	19	16.8
Wards	55	48.7
Common Places (Clinics, Corridor, Cafeteria)	39	34.5
Rotation (113)		
Obstetrics/Gynecology/Pediatrics	13	11.5
Internal Medicine	83	73.5
Surgery	17	15
Academic Level of Student (113)		
4th year (Med 1)	12	10.6
5th year (Med 2)	7	6.2
6th year (Med 3)	73	64.6
7th year (Med 4)	21	18.6
Were you alone? (113)		
Yes	46	40.7
No	67	59.3
Was the instance targeted to you alone? (113)		
Yes	69	61.1
No	44	38.9
Were you directly involved in the care of the patient? (113)		
Yes	57	50.4
No	56	49.6
Your immediate reaction (113)		
Called for help	24	21.2
Pacify	45	39.8
Explained yourself	15	13.3
Moved Away	26	23
Retaliate (Revenge)	3	2.7
Your Feeling (113)*		
Neutral	17	15
Fearful/Scared	20	17.7
Humiliated/Embarrassed	43	38.1
Angry/Sad	88	77.9
How was the Situation Addressed? (113)		
Nothing Happened	29	25.7
Another member of the team intervened	19	16.8
Superior Intervened	49	43.4
Discussion with patient, patients' family or friends	16	14.2
Was the Situation Resolved? (113)		
Yes	97	85.8
No	16	14.2
Were you satisfied with the resolution? (113)		
Yes	74	65.5
No	26	23
Wasn’t Resolved	13	11.5
Influence on Academic Performance (113)		
Decreased academic performance	40	35.4
Increased academic performance	4	3.5
Didn’t affect academic performance	69	61.1

### Association Between Mistreatment and Socio-Demographics of Medical Students

There was a significant association between mistreatment in the clinical setting and gender, type of university, and family income. Although females reported a higher incidence of mistreatment, the association was weak (*p* = 0.03, φ_c_ = 0.17). However, a moderate association was observed between mistreatment incidents and type of university, where students from private institutions reporting a higher incidence of mistreatment than those from the public institution (*p* = 0.00, φ_c_ = 0.28). Similarly, students who reported having insufficient income were more likely to experience mistreatment (*p* = 0.00, φ_c_ = 0.36). On the other hand, there was no association with nationality, place of residence, or marital status (Table 4).

**TABLE 4:** Association between mistreatment and socio-demographics of medical students (Beirut, Lebanon. 2024).

	Number (percentages)		*p*-value	Crammer’s V
Mistreatment from any source	Yes	No
	146 (48.7)	154 (51.3)		
Gender			0.03	0.174
Male	58 (39.7)	88 (57.1)		
Female	88 (60.3)	66 (42.9)		
Nationality			0.94	
Lebanese	143 (97.9)	151 (98.1)		
Non-Lebanese	3 (2.1)	3 (1.9)		
Place of Residence			0.14	
North Lebanon	18 (12.3)	13 (8.4)		
Beirut	56 (38.4)	72 (46.8)		
Bekaa-Baalbak	9 (6.2)	12 (7.8)		
Mount Lebanon	36 (27.4)	42 (27.3)		
South Lebanon	27 (18.5)	15 (9.7)		
Marital Status			0.25	
Single	136 (93.2)	148 (96.1)		
Engaged/Married	10 (6.8)	6 (3.9)		
Type of University			0.00	0.28
Public	45 (30.8)	13 (8.4)		
Private	101 (69.2)	141 (91.6)		
Family Income			0.00	0.36
Insufficient	68 (68)	27 (31)		
Sufficient	32 (32)	60 (69)		

### Knowledge of Students About Managing Mistreatment

More than half of the participants declared they did not know where to seek help in cases of mistreatment, and 64.7% said they were not informed about the ideal way to handle such instances. Three-quarters of the students believed that the person in charge should be informed; the remainder preferred filing an individual report. 61% believed the consultant in charge of the patient and the student’s clinical mentor should be involved in the debriefing while 50.3% stated the faculty members and mentors to be the best choice (Table 5).

**TABLE 5:** Knowledge of Students about Mistreatment characteristics (Beirut, Lebanon. 2024).

	Number	Percentage
Do you know where to seek help in case of mistreatment? (300)		
Yes	158	52.7
No	142	47.3
Students Informed About The Ideal Way to Handle Mistreatment (300)		
Yes	106	35.3
No	194	64.7
What do you think is the ideal way of informing the faculty of (300)		
Direct individual report	75	25
Informing the person in charge	224	74.7
Other	1	0.3
Who do you think should be involved in the debriefing? (300)^a^		
Consultant in charge of the patient & the student’s clinical mentor	183	61
Department of student affairs	89	29.7
Faculty members and mentors	151	50.3
Clerkship directors	148	49.3
Others	2	0.7

^a^ Multiple response question.

## Discussion

Medical students form the backbone of our future healthcare system; yet, literature concerning students’ mistreatment during clinical rotations in the Middle East region remains sparse. The purpose of this study is to assess the prevalence of students’ mistreatment and the knowledge of our students on mistreatment characteristics.

Half of the students reported being mistreated, which is much higher than in some regional and Western countries, particularly those with a robust healthcare infrastructure, such as Saudi Arabia (28%) [20], Turkey (36%) [21], and the United States of America (35.4%) [22]. However, higher rates were reported in Egypt (71.1%) probably due to many cultural and demographic factors [23].

Although teaching in medical school is predominantly confrontational, educators can easily come across as abusive and disparaging [24]. This is a consistent finding in many countries around the world such as Canada, Pakistan, and Chile [25–27], and also was reported in our study. This study also highlighted a major source of mistreatment by patients and their families/friends which were highly prevalent (77.4%), in comparison to other countries like Singapore (14.3%) for example [19]. Most of these incidents occurred during history taking and mainly took place during internal medicine rotations, particularly in wards which may be due to a high level of student-patient interaction.

In comparison to other studies, medical students in their senior years fell victim to more offenses, likely due to increased time they spent in the clinical setting [19]. More than half of the medical students were reported to be directly involved in patient care, which reinforces the notion that the patients and their families perceive the medical students as being incompetent, less experienced, and less skilled in handling their care.

Regarding students’ emotions, the majority of them felt angry/sad, yet around 15% of them felt neutral. This may be due to either students having been immunized or used to it, or students not caring about patients' perception, the latter being alarming. Most of the mistreated students, pacified themselves and did nothing, and only 21.2% decided to call for help, reflecting a lack of knowledge about handling techniques during these situations or the presence of intimidating barriers preventing students from standing up for their rights. An alarming sign is that 2.7% of victims chose to retaliate, which is capable of creating a dangerous situation of tension in which the patient’s wellbeing is at risk. While most of the students claimed that this incident did not affect their academic performance, 35.4% claimed it decreased their academic performance. Further studies, however, are required to assess the impact of these offenses on their mental wellbeing.

Females were subjected to more mistreatment incidents, probably because our society is so used to men dominating STEM (science, technology, engineering, and math) [28, 29]. The majority of mistreated individuals were enrolled in private universities, most of which are affiliated with private hospitals in which patients covered directly or indirectly (through medical insurance) their medical expenses, thus tolerated less student involvement in their medical care.

The majority of participants were not trained about mistreatment incidents or characteristics, and 47.3% of them did not know where to seek help. These results show the need for faculties and hospitals to spread awareness among medical students about the proper handling of such situations.

While mistreatment as some participants suggested, is bound to happen and unavoidable, it is essential to also clear up any misconceptions or discomfort patients may have about training medical students involved in their care [30, 31]. Most of the students believed reporting to the person in charge is the ideal action to take, especially if the insults come from patients. However, most of the students might not be eager to blow the whistle to avoid any harm when educators (attending physicians and residents) were the offenders. The American Association of Medical Colleges (AAMC) distributes a Graduation Questionnaire that contains a dedicated section to collect feedback about their experiences during medical school and their clinical practice [32]. Providing medical students with such an anonymous survey at the end of each course is an important way to tackle this issue. It not only reassures students about the protection of their identity but also is an effective way of collecting reports about mistreatment incidents and their effect on students’ wellbeing.

Multiple studies have suggested that medical student mistreatment has been associated with stress, depression, suicidality, and alcohol binge drinking [9, 24]. Repeated encounters with such mistreatment may spike anxiety and emotional distress, which may affect the future performance of students [9]. It also diminishes students’ enthusiasm for learning the curriculum, which decreases the knowledge that students acquire by the end of their education, negatively impacting patients’ outcomes down the line [9].

### Conclusion

Medical student mistreatment is highly prevalent in Lebanon, with patients, and educators (attending physicians and residents) being the most significant sources. Such mistreatment has a significant impact on students and further studies are required to measure the extent of this impact on their mental health.

### Limitations

This is a cross-sectional study; therefore, no causalities can inferred. Recall bias may be present due to the data collection procedure. There is no information about the severity of mistreatment and how students define and classify mistreatment.
